# Endocrine therapy resistance: what we know and future directions

**DOI:** 10.37349/etat.2022.00096

**Published:** 2022-08-31

**Authors:** David Musheyev, Anya Alayev

**Affiliations:** Alayev Lab, Stern College for Women, Biology Department, Yeshiva University, New York, NY 10174, USA; University of Aberdeen, UK

**Keywords:** Endocrine resistance, estrogen receptor-positive, signaling crosstalk

## Abstract

Endocrine resistance is a major hurdle in the treatment of estrogen receptor (ER)-positive breast cancer. When abnormally regulated, molecular signals responsible for cellular proliferation, as well as ER itself, allow for cellular evasion of ER-dependent treatments. Therefore, pharmacological treatments that target these evasion mechanisms are beneficial for the treatment of endocrine-resistant breast cancers. This review summarizes currently understood molecular signals that contribute to endocrine resistance and their crosstalk that stem from mitogen-activated protein kinase (MAPK), phosphoinositol-3 kinase/protein kinase B (PI3K/AKT), mechanistic target of rapamycin (mTOR), cyclin-dependent kinases 4 and 6 (CDK4/6) and aberrant ER function. Recent clinical trials that target these molecular signals as a treatment strategy for endocrine-resistant breast cancer are also highlighted.

## Introduction

The connection between the function of endocrine organs and breast cancer was described as early as 1896 in a case series by Beatson [1], who described remission of breast cancer after a bilateral oophorectomy, an excision of ovaries, in premenopausal women. Because the women described were premenopausal, their ovaries were likely still producing estrogen until their removal. The removal of the estrogen-producing organs could be correlated with the remission of cancer implying cancer’s sensitivity to steroid hormones such as estrogen. Beatson’s observations came 62 years before the discovery of the estrogen receptor (ER) by Fuentes and Silveyra [2] and its eventual correlation with breast cancer in future studies. Known to be sensitive to estrogen signaling due to its expression of the ER, ER-positive breast cancer accounts for 75% of all breast cancer [3]. Discoveries in the signaling of ER and its effects on breast cancer led to different therapeutic approaches to treating ER-positive breast cancer by targeting ER. These treatments include selective ER modulators (SERMs), selective ER down-regulators (SERDs), and aromatase inhibitors (AIs), all of which have been shown to reduce mortality and disease recurrence, and were followed by the development of combination therapies [4]. However, resistance to these therapies poses a challenge and requires alternative treatments. Mechanisms of resistance stem from two major sources, direct effects on ER—such as mutations and alterations in functions—or through activation of parallel molecular signals leading to ER independent cellular proliferation.

## ER

### Structural domains

**Figure 1.**
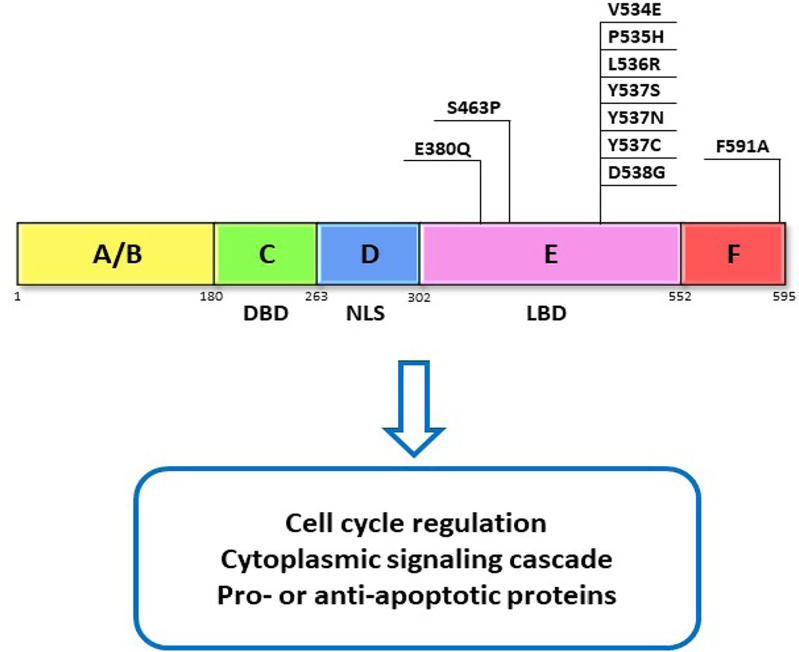
ER domains and function. ERα functional domains and functions are represented as A–F. DBD, NLS, and LBD domain as well as their commonly reported mutations in the LBD. These mutations have been found to contribute to resistance through activation of ERα which is independent of its ligand

The ER is a transcription factor that contains 6 functional domains termed by letters A–F that represent relate to specific functions of ER. Most notably, the C domain contains the DNA binding domain (DBD) that allows for ER’s nuclear action, the D domain contains nuclear localization signal (NLS) that allows translocation of ER to the nucleus, and the E domain contains the ligand binding domain (LBD) that allows interaction of ER with its ligand—estrogen (Figure 1) [5]. There are two ERs, ERα and ERβ, that share homology at the protein level [5], however, they differ in their cellular actions and are variably expressed in different tissue types [6]. Because it is the ERα subtype that is predominately expressed in breast cancers and has been targeted by endocrine therapies in the treatment of breast cancer, our discussion will focus on targeting Erα [6–8].

### Signaling

Effects from ERα signaling can follow canonical pathways, whereby the ERα effect is nuclear and acts either directly or through intermediate activators and promoters on genetic response elements. Alternatively, the effect can be non-canonical, and exhibit immediate cytoplasmic response in cellular signaling cascades such as through phosphorylation of extracellular signal-regulated kinase (ERK) or protein kinase B (AKT) [9]. Transcriptional effects of ERα may be manifested either through canonical or non-canonical pathways, which is known as “nuclear-initiated steroid signaling” (NISS) [10]. Such nuclear functions through direct ER actions involve the cytoplasmic binding of estrogen to ER and the translocation of ER into the nucleus to act on enhancers and promoters that contain particular palindromic sequences known as estrogen response elements (EREs) [11–13]. Such actions depend on transcription and translation and therefore tend to be slower than the direct-acting membrane-initiated steroid signaling (MISS), which is activated by membrane-bound ER and leads to the activation of kinase signaling cascades [7]. Signaling pathways just downstream of MISS include a variety of signaling cascades including mitogen-activated protein kinase (MAPK), AKT, and Src [7, 14]. Furthermore, ER activation can occur independently of its known ligand, estrogen, through the ligand-independent mechanism, whereby ER is phosphorylated directly by other signaling cascades such as the MAPK/ERK pathway [15] or the phosphoinositol-3 kinase (PI3K)/AKT pathway [16, 17]. Responses to both nuclear and membrane-initiated signaling, as well as ligand-independent signaling, have been implicated in tumorigenesis and discoveries in the signaling pathways have allowed for the development of combination therapies and more targeted treatment [18]. Convergence between canonical and non-canonical ER activation as well as crosstalk between nuclear initiated and membrane initiated signaling has been implicated in treatment resistance and has been a topic of investigation in clinical and pre-clinical studies [19–21].

## Endocrine therapies

Treatments targeting estrogen signaling either through a reduction in estrogen production or direct action on the receptor are known as endocrine therapies and have been a part of the core strategy of treating ER-positive breast cancer. SERDs and SERMs both act directly on ER and reduce signaling with slight variations in their mechanisms of action. While SERMs compete with estrogen for receptor binding, SERDs bind with ER and mark ER for degradation [22]. Despite also being an endocrine therapy that affects ER, AI action does not involve direct action with ER, however, it reduces estrogen production by inhibiting a key step in the conversion of testosterone to estradiol [23].

### SERMs

The first Food and Drug Administration (FDA)-approved endocrine therapy was in the 1970s when tamoxifen, a SERM, has become the mainstay therapy in the treatment of ER-positive breast cancer and has been shown to reduce mortality and recurrence risk for 15 years after 5 years of treatment [24]. Despite being relatively well tolerated, some patients experienced adverse side effects and lowering the dose and duration of tamoxifen in patients with ER-positive breast cancer in an attempt to reduce side effects still yielded benefits in overall survival and a reduced recurrence rate [25]. In the ATLAS trial, in patients who tolerated treatment, intervention with tamoxifen monotherapy in early-stage breast cancer for 10 years improved overall survival and late recurrence when compared to 5 years of therapy, further highlighting the impactful utility of SERMs in endocrine sensitive tumors [26].

### SERDs

Despite having similar mechanisms of action to SERMs, SERDs bind to ERα with a stronger affinity than tamoxifen [27] and were shown to be effective in both tamoxifen-resistant and tamoxifen-sensitive cell lines [28]. Unlike SERMs, they are primarily approved for use in postmenopausal patients [29].

### AIs

Similar to SERDs, AIs were found to provide a better clinical response rate in postmenopausal women when compared to women treated with tamoxifen [30]. In particular, the third generation AI, letrozole, has been shown to increase the time to disease progression when compared to tamoxifen [31].

### Luteinizing hormone-releasing hormone analogues

In an attempt to identify alternative targets for treating breast cancers, exploratory studies looked for hormone receptors beyond the classic three targets in breast cancer. A receptor that was identified to be present in at least half of their samples was luteinizing hormone-releasing hormone (LHRH) and was even identified in triple-negative breast cancer samples, that are notoriously resistant to traditional targeted hormone treatments [32, 33]. Leuprolide is an LHRH agonist that, when given continuously inhibits pituitary hormone release (“chemical oophorectomy”) and leads to a subsequent reduction of estrogen production—similar to that described in Beatson’s oophorectomies. For this reason, it is primarily used to treat premenopausal women with limited use in hormone-resistant cancers [34]. Younger patients tend to have a higher risk of recurrence, therefore ovarian function suppression with gonadotrophin-releasing hormone (GnRH) agonists such as leuprolide. Treatment with LHRH agonists can preserve ovarian function by reversibly suppressing ovarian function and can rescue function post-chemotherapy, even in hormone receptor-negative tumors [35]. This strategy of preserving ovarian function is especially important to younger patients who have cancers dependent on chemotherapy, which can have detrimental effects on fertility [36]. It is important to note, that this restoration of ovarian function in some instances can even occur post-menopause. In a case report, a patient was described to have a resumption of menses post-AI treatment despite having previously been determined to be postmenopausal, suggesting a rescue of ovarian function by AI and necessitating closer monitoring of patient status and fertility and appropriate patient education [37].

### Need for alternative therapies

Despite the three classes of endocrine therapies’ action on estrogen signaling, the difference between the therapies in the patient population they benefit emphasizes the importance of tailoring treatments to the disease. In the case of hormone therapy treatment resistance, switching to different hormone therapies is a strategy that is implemented in an attempt to overcome resistance. Unfortunately, response rates to treatment after switching over are not always as impressive as they are with initial hormone treatments, and require different treatment strategies [38]. For example, in patients who developed resistance to tamoxifen therapy, crossover to AI therapy reduced the risk of recurrence, however, did not provide a survival benefit [24]. Indicating that we still need a better understanding of the mechanisms that contribute to endocrine resistance and the development of better treatment strategies to overcome this resistance.

## Endocrine therapy resistance

Resistance to endocrine therapies can be acquired as a result of long-term endocrine therapy or be a result of an intrinsic mechanism at play before the initiation of treatment. Both of these mechanisms can stem from direct actions on ER, such as the case of estrogen receptor 1 (*ESR1*) mutations leading to ER hyperactivation or to activation of ER-independent pathways responsible for cell growth and survival.

### *ERα* mutations

About 20% of ER-positive breast cancers lose ER expression and switch over to ER-independent mechanisms for cell proliferation after treatment with endocrine therapies [39, 40]. Examples of the loss of ER expression can stem from histone deacetylase inhibition [41] or methylation at the *ESR1* gene promoter [42]. Direct mutations in the *ESR1* gene can also lead to resistance. For instance, mutations in the LBD (Figure 1), which are found in 20% of metastatic ER-positive cancers and are often acquired following hormone treatment [43–48]. Such mutations cause ER to take on a conformation similar to the estrogen-bound receptor and therefore lead to constitutive activation of ER signaling independent of any ligands [49]. Some mutations can alter the transcriptional profile of genes activated in response to ER signaling leading to activation of pathways involved in growth, invasion, and metastasis, thereby creating a more aggressive tumor that is less responsive to conventional therapies [50, 51]. An even rarer mutation results in the fusion of *ESR1* to different genes leading to constitutive activation of ER target genes and estrogen-independent growth [52]. Identification of specific mutations might offer avenues for creating treatments based on the specific mutations, as was identified in the case of ESR1-fusions, which were highly sensitive to treatment with a SERM/SERD and human epidermal growth factor receptor 2 (HER2) or Src inhibitor and cyclin-dependent kinases 4 and 6 (CDK4/6) inhibitors [53, 54]. All of these changes to ER expression in one way or another lead to hyperactivation of ER that is independent of estrogen. This makes endocrine therapy a less useful intervention in the treatment of these tumors as reducing the production of estrogen, in the case of AIs, or competing for ER binding sites, with SERMs, would not change the signaling outcome. Though next-generation SERMS and SERDs are able to act on ER despite certain mutations [55] and have shown to be relatively tolerable in phase 1 studies [56, 57], these treatments do not address the activation of other pro- survival signaling mechanisms.

### Non-*ER* mutations

Studies characterizing molecular mechanisms responsible for tumorigenesis in breast cancer highlight the vast heterogeneity in breast cancers and elucidate potential mechanisms that are responsible for treatment resistance as well as offer treatment strategies to overcome resistance [58, 59]. Such studies identified clusters of changes in cells including mutations in ERα, mutations in the rat sarcoma virus/Raf/extracellular-signal regulated kinase kinases/MAPK (Ras/Raf/MEK/MAPK) pathway, as well as other molecular pathways and transcription factors [4]. More frequently occurring mutations that lead to the loss of function in the corepressors of ER, and subsequent endocrine resistance, occur in 13– 55% of breast cancers and lead to disinhibition of ER expression. However, preclinical studies show an acquired sensitivity to combination treatment with histone deacetylase inhibitors, thus further highlighting the importance of tumor characterization [17]. The above-mentioned mutations lead to a shift towards ER-independent signaling, essentially making the tumor behave as if it were ER-negative. Treatments targeting these alternatively activated pathways would be more appropriate in these cases than traditional endocrine therapy.

### Crosstalk between signaling pathways

Parallel signaling pathways that regulate cell proliferation, protein synthesis, and metabolism, may interact with ER signaling and with other pathways and may contribute to the progression of cancer, despite endocrine therapy. This is because an alternative mechanism is available for estrogen signaling that is independent of direct ligand binding with ER as a result of cytoplasmic crosstalk.

Both independent of estrogen signaling and through the reciprocal activation of ER bidirectional crosstalk between growth factor signaling cascades and ER lead to the activation of downstream pro-survival signaling [60, 61]. Examples of such estrogen-independent signaling include ER activation through ERK1/2 and PI3K/AKT, which phosphorylate ER on serines 118 and 167 causing resistance to endocrine therapy [15, 62]. Often, such tumors would exhibit an increase in the expression and activity of growth factors driven by several causes including activating mutations [4] and loss of ER signaling [63]. Such growth factor-induced ER signaling, interestingly, exhibits a distinct expression profile that differs from estrogen-dependent ER signaling [64]. Such a shift in growth factor overactivation, particularly fibroblast growth factor receptor (FGFR), was observed in 40% of metastatic ER-positive breast cancer after acquiring resistance to endocrine therapy and is associated with a worse prognosis [65, 66]. The estrogen-independent ER activation resulting from signal crosstalk underscores the importance of targeting multiple pathways, including the ER, in the case of treatment resistance and the importance of tumor categorization to identify the appropriate polytherapy.

## Strategies to overcome resistance

Studies showing an improvement in breast cancer survival in response to implementing community cancer screening and subsequent earlier detection and treatment of breast cancer suggest a correlation between treatment success and relative time of treatment in the disease course [67]. The earlier treatment is less likely to be endocrine-resistant, and it is important to not only treat early in the disease course but also treat appropriately to the tumor type and molecular profile. Due to the crosstalk between ER and other signaling pathways, novel treatment strategies are aimed at inhibiting signaling pathways related to receptor tyrosine kinases, including MAPK and PI3K/AKT, and CDK4/6. In the case of hyperactivation of growth factor signaling, treatments that target tyrosine kinases and other downstream effectors of the growth factors were shown to improve the sensitivity to endocrine therapy [68, 69], and offer an avenue for polytherapy approaches in targeting multiple pathways simultaneously in response to acquired resistance. For example, in ER-positive cells with HER2 activating mutations, targeting HER2 or downstream effectors such as PI3Kα and target of rapamycin complex 1 (TORC1) sensitized the cells to fulvestrant treatment [70]. Clinically, combination treatment with endocrine therapy and HER2 inhibitors in patients with ER-positive breast cancer and HER2 activating mutations led to improved progression-free survival and duration of response when compared to endocrine therapy alone, thus showing benefit in combination approaches based on tumor characteristics [71].

### Receptor tyrosine kinases and associated molecular pathways

Dysregulation in receptor tyrosine kinase signaling has long been implicated in the development of cancer due to its involvement in a variety of key cellular functions including growth, differentiation, and motility [72]. Particularly in breast cancer, upregulation of tyrosine kinase receptors has been correlated with a poor prognosis [73–75]. Tyrosine kinase receptor signaling starts at the cellular membrane where the receptor is activated through an auto-phosphorylation event, commonly through direct extracellular ligand-binding by growth factors which leads to further downstream activation of kinase cascades including PI3K/AKT and MAPK [76, 77]. These cascades then activate cell growth and survival pathways [78, 79]. Dysregulation of elements along these signaling pathways, either through activating mutations, upregulation of activators, or pathway crosstalk, drives the pathogenesis of cancer by promoting cell proliferation [78, 80]. Dysregulation of receptor tyrosine kinase signaling has been correlated with endocrine resistance and combination therapies with endocrine therapy and receptor tyrosine kinase inhibitors have yielded promising results in preclinical studies by reversing endocrine resistance and inducing apoptosis [81]. Pathways downstream of receptor tyrosine kinases that are often implicated in endocrine resistance and have been investigated for the treatment of endocrine-resistant breast cancer include the MAPK signaling pathway and the PI3K/AKT pathways as well as their shared downstream mechanistic target of rapamycin (mTOR) complex and CDK4/6.

### MAPK

**Figure 2.**
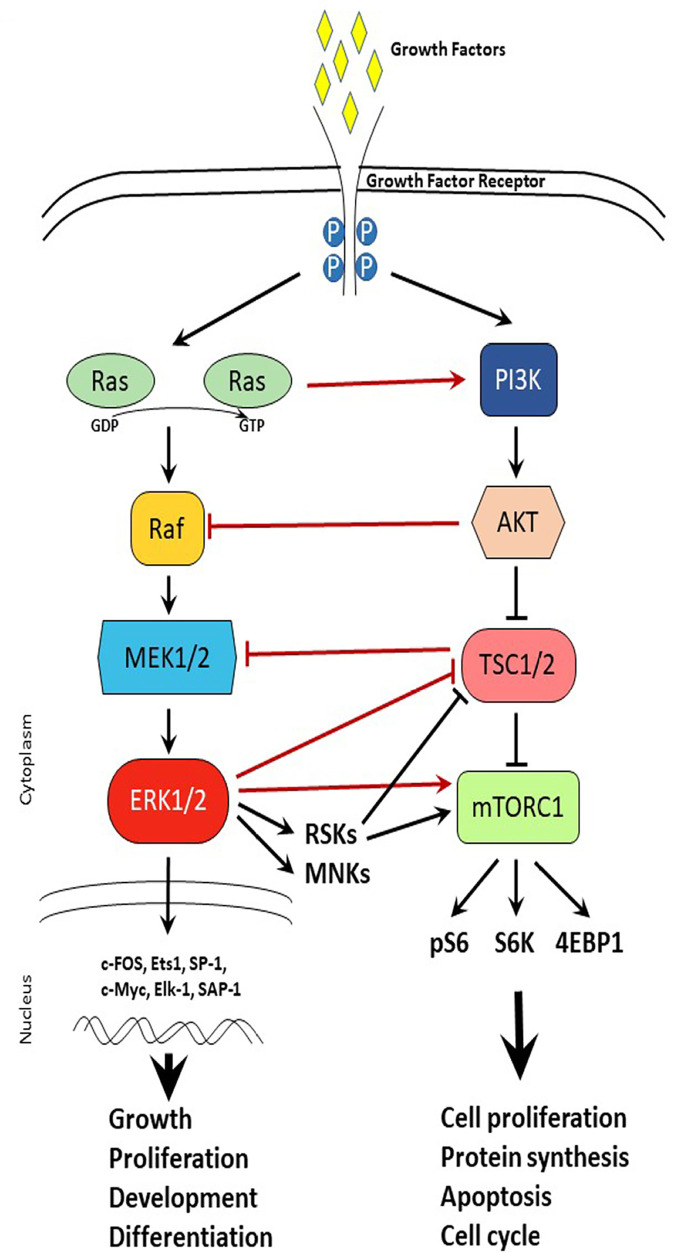
Cross-talk between MAPK and mTOR signaling pathways. Schematic representation of MAPK and mTOR signaling pathways upon growth factor activation. Arrows represent activation, while t-shaped lines represent inhibition. Black lines depict signaling within a pathway. Red lines depict crosstalk between signaling pathways. TSC1/2: tuberous sclerosis 1/2; 4EBP1: eukaryotic translation initiation factor 4E-binding protein 1; P: phosphorylation; pS6: phosphorylated S6; GDP: guanosine diphosphate; GTP: guanosine triphosphate

MAPK signaling pathway is activated by growth factors and regulates cellular processes such as growth, proliferation, development, and differentiation. The MAPK pathway includes cascades of serine-threonine kinases with three major pathways: ERK, p38, and c-Jun [82]. Upon growth factor binding to the growth factor receptor, the Ras/Raf/MEK1/2 activation leads to activation of ERK1/2 in the cytoplasm. In turn, ERK1/2 can have cytoplasm action via activation of downstream targets such as MAPK interacting protein kinases (MNKs) and ribosomal S6 kinases (S6Ks, RSKs) or nuclear action via activation of transcription factors such as c-FOS, c-Myc, and activating protein-1 (AP-1) (Figure 2). Overactivation of the MAPK signaling pathway has been shown to be a driver of tamoxifen resistance and treatments with drugs targeting the signaling cascade were successful in reversing the resistance [65, 83]. Of these pathways, the ERK pathway is known to act on ERα by phosphorylating it on serine 118 leading to estrogen-independent ER activation [84]. Inhibition of the ERK pathway has been shown to upregulate ERα and sensitize cancer cells to tamoxifen [85]. Similarly, another experiment in MCF-7 cells that overexpress epidermal growth factor receptor (EGFR) showed that ERK inhibition sensitized the cells to SERD treatment [43]. This evidence highlights the crosstalk between ER signaling and ERK signaling and suggests the potential for a self-perpetuating positive loop between the two pathways [86]. Another way that estrogen signaling affects the MAPK pathway is through G protein-coupled ER (GPER)-dependent EGFR activation and related pathways [87, 88]. GPER is thought to be an ER-related protein that is membrane-bound and is similarly activated by estrogen [89, 90]. In an ER-negative cell line estrogen treatment after transfection with GPER led to an increase in MAPK phosphorylation, an effect that was not observed in cells that were not expressing GPER [91]. Furthermore, extended treatment with tamoxifen led to an increase in membrane-bound GPER, suggesting a potential alternative ER-independent mechanism that can be activated with estrogen [92].

### PI3K/AKT/mTOR

The mTOR signaling pathway is a master sensor of nutrient availability in the cell that regulates processes such as cell proliferation, protein synthesis, apoptosis, and cell cycle. Just downstream of the PI3K/AKT pathway, mTOR can form two different complexes that are distinctly known as mTOR complex 1 (mTORC1) and mTORC2 [93, 94]. mTORC1 is composed of mTOR, raptor, deptor, and LST8 and is known to regulate cell growth, protein synthesis, autophagy, and metabolism [95, 96]. Activation of mTOR promotes cell proliferation which is seen in endocrine- resistant breast cancer [97]. mTOR is also upregulated by ERK and PI3K/AKT pathways, which poses an additional layer of signaling that is stimulating tumor survival and contributing to treatment resistance, however, offers an additional co-target to consider in combination therapies [98, 99]. Just downstream of mTORC1, S6K1 is often amplified in breast cancer and is correlated with a poorer prognosis [100]. Furthermore, S6K1 action on ERα also leads to a positive feedback loop just as observed with mTORC1, whereby S6K1 phosphorylation of ER leads to ER activation and, through mTOR, ER action activates S6K1 [48, 101]. Such positive feedback loops highlight the importance of combinatorial therapies to break the feedback signaling (Figure 2).

Evidence of crosstalk between the PI3K/AKT and MAPK and involvement of other tumorigenic pathways, as was seen with mTOR, has been identified in numerous studies. This crosstalk can sometimes be missed in patients as was seen with the case of acquired HER2 activation with endocrine resistance despite an initial negative HER2 status. Interestingly, HER2 activation was previously described in acquired endocrine resistance despite initial low expression and is understood to be a result of crosstalk between multiple pathways including MAPK and PI3K/AKT [102]. This crosstalk might explain the treatment response to trastuzumab, a HER2 inhibitor in patients whose tumors were initially expressing low levels of HER2 [103].

### CDK4/6

Further downstream of the receptor tyrosine kinase signaling and of ER, the activation of CDKs contributes to tumor proliferation and cell cycle progression as a result of transcriptional upregulation of cell cycle regulators, these include CDK2 and CDK4 [104]. Signaling responsible for CDK4/6 upregulation includes upstream mitogenic pathways such as PI3K/AKT, MAPK, mTOR, and ER signaling [105]. In the case of endocrine resistance, these CDKs are often found to be aberrantly hyperactivated [106]. Palbociclib, ribociclib, and abemaciclib are currently FDA-approved CDK4/6 inhibitors and are used in combination with endocrine therapies for the treatment of ER-positive/HER2-negative breast cancer [107, 108]. In the PALOMA-2 trial, combination therapy with palbociclib and letrozole led to improvements in progression-free survival compared to letrozole monotherapy in patients with untreated ER-positive/HER2-negative breast cancer [107]. Similar results were achieved in the PALOMA-3 trial in patients who had disease progression after initial treatment failure from endocrine therapy [108]. The discovery of crosstalk between CDK4/6 activity and signaling cascades that involve PI3K/mTOR [109] and FGFR [110] has sparked further studies that propose the use of triple therapy with CDK4/6 inhibitors, endocrine therapy, and drugs targeting the respective signaling cascades. In the TRINITI-1 trial combination treatment with a CDK4/6 inhibitor and everolimus in addition to endocrine therapy had the overall benefit and was relatively well tolerated [111].

## Novel clinical approaches for treatment of endocrine resistant breast cancer

Taking into account the molecular pathways involved in endocrine resistance and the dynamic nature of disease progression, clinical trials investigating treatment options in endocrine-resistant breast cancers turn to combinatory treatments and crossover to different therapies in response to shifts in molecular signaling. Combinatory treatments targeting pathways downstream of receptor tyrosine kinases, mTOR, and CDK4/6 have shown promise in clinical trials.

Recent trials investigating the clinical benefit of inhibition of CDK4/6 yielded favorable results [112]. Results from the MONARCH 2 trial, a phase 3 randomized controlled trial (RCT) in which patients with ER-positive/HER2-negative breast cancer were treated with fulvestrant in combination with abemaciclib, CDK4/6 inhibitor, or with fulvesterant with placebo, resulted in improvements in overall survival in the abemaciclib treated group compared to the placebo-treated group [113]. Similarly the phase 2, LETPAL trial showed the benefit of the CDK4/6 inhibitor palbociclib with letrozole combination therapy when compared to chemotherapy in ER-positive/HER-negative breast cancer [114]. The results from these clinical trials further solidify the evidence found in pre-clinical investigations that were discussed earlier, showing that targeting CDK4/6 in conjunction with ER allows us to circumvent a cellular adaptation that causes upregulation in CDK4/6 and its resulting endocrine resistance. We now know that crosstalk among multiple pathways can contribute to treatment resistance, therefore studies are further investigating the use of polytherapies that target the known players in endocrine resistance. In a study investigating triple intervention with a CDK4/6 inhibitor, AKT inhibitor, and endocrine therapy in resistant tumors in postmenopausal ER-positive/HER2-negative breast tumors, initial results of the phase 1 trial showed that the triple therapy was well tolerated [115].

Evidence of the involvement of receptor tyrosine kinases and their downstream serine-threonine kinases in endocrine resistance prompted further investigations and eventually led to the characterization of the *PIK3CA* mutation which is found in nearly 40% of ER-positive breast cancers [116, 117]. The recent SOLAR-1 trial investigate a PI3K inhibitor, alpelisib, and found a benefit in median overall survival among patients with the *PIK3CA* mutation who were treated alpelisib and fulvesterant when compared to patients treated with fulvesterant and placebo [118]. This correlation with tumor characterization with its matching treatment allows for more effective treatment strategies than targeting ER alone.

Another receptor tyrosine kinase implicated in endocrine resistance that is currently investigated is the HER2 receptor. In endocrine-resistant breast cancer with low HER2 expression, treatment with an antibody-drug conjugate has shown promising results in clinical and preclinical studies [119]. Suggesting possible signaling overlap between HER2 and other members in the EGFR family of receptor tyrosine kinases. Trials investigating the transition to activating other molecular pathways give us a better understanding of the evolution of tumors as they progress through different treatments and offer insight into the value of retyping tumors as they progress.

## Conclusions

The multitude of molecular subtypes of breast cancers highlights the heterogeneity of the disease and the need for disease classification and the development of tailored therapies. Furthermore, molecular changes throughout treatment as a result of genetic changes and crosstalk between multiple signaling cascades elucidates the dynamic nature of breast cancer progression and the value of polytherapy to simultaneously target multiple pathways to circumvent the effects of future shifts in molecular signaling. In response to this heterogeneity and dynamic molecular shifts technologies to assay tumor types have been developed to improve tumor classification and implement more targeted treatments [120]. The results of these studies identify specific targets in molecular signaling and mutations that may be used in the future to predict endocrine resistance prior to a trial of endocrine therapy, therefore allowing for a more aggressive treatment earlier in their disease course.

## Abbreviations

AIs: aromatase inhibitors
AKT: protein kinase B
CDK4/6: cyclin-dependent kinases 4 and 6
DBD: DNA binding domain
EGFR: epidermal growth factor receptor
ER: estrogen receptor
ERK: extracellular signal-regulated kinase
ESR1: estrogen receptor 1
GPER: G protein-coupled estrogen receptor
HER2: human epidermal growth factor receptor 2
LBD: ligand binding domain
LHRH: luteinizing hormone-releasing hormone
MAPK: mitogen-activated protein kinase
mTOR: mechanistic target of rapamycin
mTORC1: mechanistic target of rapamycin complex 1
NLS: nuclear localization signal
PI3K: phosphoinositol-3 kinase
S6Ks: S6 kinases
SERDs: selective estrogen receptor down-regulators
SERMs: selective estrogen receptor modulators

## Declarations

### Copyright

© The Author(s) 2022.

## References

[1] Beatson GT. On the treatment of inoperable cases of carcinoma of the mamma: suggestions for a new method of treatment, with illustrative cases. Trans Med Chir Soc Edinb. 1896;15:153–79. 29584099 PMC5518378

[2] Fuentes N Silveyra P. Estrogen receptor signaling mechanisms. Adv Protein Chem Struct Biol. 2019;116:135–70. 10.1016/bs.apcsb.2019.01.001 31036290 PMC6533072

[3] DeSantis CE Ma J Gaudet MM Newman LA Miller KD Goding Sauer A Breast cancer statistics, 2019. CA Cancer J Clin. 2019;69:438–51. 10.3322/caac.21583 31577379

[4] Razavi P Chang MT Xu G Bandlamudi C Ross DS Vasan N The genomic landscape of endocrine-resistant advanced breast cancers. Cancer Cell. 2018;34:427–38.e6. 10.1016/j.ccell.2018.08.008 30205045 PMC6327853

[5] Kumar V Green S Stack G Berry M Jin JR Chambon P. Functional domains of the human estrogen receptor. Cell. 1987;51:941–51. 10.1016/0092-8674(87)90581-2 3690665

[6] Anderson WF Chatterjee N Ershler WB Brawley OW. Estrogen receptor breast cancer phenotypes in the surveillance, epidemiology, and end results database. Breast Cancer Res Treat. 2002;76:27–36. 10.1023/A:1020299707510 12408373

[7] Pedram A Razandi M Blumberg B Levin ER. Membrane and nuclear estrogen receptor α collaborate to suppress adipogenesis but not triglyceride content. FASEB J. 2016;30:230–40. 10.1096/fj.15-274878 26373802 PMC4684544

[8] Langdon SP Herrington CS Hollis RL Gourley C. Estrogen signaling and its potential as a target for therapy in ovarian cancer. Cancers (Basel). 2020;12:1647. 10.3390/cancers12061647 32580290 PMC7352420

[9] Musgrove EA Sutherland RL. Biological determinants of endocrine resistance in breast cancer. Nat Rev Cancer. 2009;9:631–43. 10.1038/nrc2713 19701242

[10] Veetil AT Jani MS Krishnan Y. Chemical control over membrane-initiated steroid signaling with a DNA nanocapsule. Proc Natl Acad Sci U S A. 2018;115:9432–7. 10.1073/pnas.1712792115 29531078 PMC6156627

[11] Scheidereit C Krauter P von der Ahe D Janich S Rabenau O Cato AC Mechanism of gene regulation by steroid hormones. J Steroid Biochem. 1986;24:19–24. 10.1016/0022-4731(86)90026-9 3009974

[12] Klein-Hitpass L Ryffel GU Heitlinger E Cato AC. A 13 bp palindrome is a functional estrogen responsive element and interacts specifically with estrogen receptor. Nucleic Acids Res. 1988;16:647–63. 10.1093/nar/16.2.647 3340549 PMC334683

[13] Gruber CJ Gruber DM Gruber IM Wieser F Huber JC. Anatomy of the estrogen response element. Trends Endocrinol Metab. 2004;15:73–8. 10.1016/j.tem.2004.01.008 15036253

[14] Micevych PE Wong AM Mittelman-Smith MA. Estradiol membrane-initiated signaling and female reproduction. Compr Physiol. 2015;5:1211–22. 10.1002/cphy.c140056 26140715 PMC4714864

[15] Kato S Endoh H Masuhiro Y Kitamoto T Uchiyama S Sasaki H Activation of the estrogen receptor through phosphorylation by mitogen-activated protein kinase. Science. 1995;270:1491–4. 10.1126/science.270.5241.1491 7491495

[16] Simoncini T Hafezi-Moghadam A Brazil DP Ley K Chin WW Liao JK. Interaction of oestrogen receptor with the regulatory subunit of phosphatidylinositol-3-OH kinase. Nature. 2000;407:538–41. 10.1038/35035131 11029009 PMC2670482

[17] Légaré S Basik M. Minireview: the link between ERα corepressors and histone deacetylases in tamoxifen resistance in breast cancer. Mol Endocrinol. 2016;30:965–76. 10.1210/me.2016-1072 27581354 PMC5414611

[18] Jeffreys SA Powter B Balakrishnar B Mok K Soon P Franken A Endocrine resistance in breast cancer: the role of estrogen receptor stability. Cells. 2020;9:2077. 10.3390/cells9092077 32932819 PMC7564140

[19] Björnström L Sjöberg M. Mechanisms of estrogen receptor signaling: convergence of genomic and nongenomic actions on target genes. Mol Endocrinol. 2005;19:833–42. 10.1210/me.2004-0486 15695368

[20] Osborne CK Shou J Massarweh S Schiff R. Crosstalk between estrogen receptor and growth factor receptor pathways as a cause for endocrine therapy resistance in breast cancer. Clin Cancer Res. 2005;11:865s–70s. 10.1158/1078-0432.865s.11.2 15701879

[21] Schiff R Massarweh SA Shou J Bharwani L Mohsin SK Osborne CK. Cross-talk between estrogen receptor and growth factor pathways as a molecular target for overcoming endocrine resistance. Clin Cancer Res. 2004;10:331S–6S. 10.1158/1078-0432.CCR-031212 14734488

[22] Patel HK Bihani T. Selective estrogen receptor modulators (SERMs) and selective estrogen receptor degraders (SERDs) in cancer treatment. Pharmacol Ther. 2018;186:1–24. 10.1016/j.pharmthera.2017.12.012 29289555

[23] Dowsett M Howell A. Breast cancer: aromatase inhibitors take on tamoxifen. Nat Med. 2002;8:1341–4. 10.1038/nm1202-1341 12457166

[24] Early Breast Cancer Trialists' Collaborative Group (EBCTCG) Davies C Godwin J Gray R Clarke M Cutter D Darby S Relevance of breast cancer hormone receptors and other factors to the efficacy of adjuvant tamoxifen: patient-level meta-analysis of randomised trials. Lancet. 2011;378:771–84. 10.1016/S0140-6736(11)60993-8 21802721 PMC3163848

[25] DeCensi A Puntoni M Guerrieri-Gonzaga A Caviglia S Avino F Cortesi L Randomized placebo controlled trial of low-dose tamoxifen to prevent local and contralateral recurrence in breast intraepithelial neoplasia. J Clin Oncol. 2019;37:1629–37. 10.1200/JCO.18.01779 30973790 PMC6601429

[26] Davies C Pan H Godwin J Gray R Arriagada R Raina V Adjuvant Tamoxifen: Longer Against Shorter (ATLAS) Collaborative Group. Long-term effects of continuing adjuvant tamoxifen to 10 years *versus* stopping at 5 years after diagnosis of oestrogen receptor-positive breast cancer: ATLAS, a randomised trial. Lancet. 2013;381:805–16. Erratum in: Lancet. 2013;381:804. Erratum in: Lancet. 2017;389:1884. 23219286 10.1016/S0140-6736(12)61963-1 PMC3596060

[27] Wakeling AE. Similarities and distinctions in the mode of action of different classes of antioestrogens. Endocr Relat Cancer. 2000;7:17–28. 10.1677/erc.0.0070017 10808193

[28] Lykkesfeldt AE Madsen MW Briand P. Altered expression of estrogen-regulated genes in a tamoxifen-resistant and ICI 164,384 and ICI 182,780 sensitive human breast cancer cell line, MCF-7/TAMR-1. Cancer Res. 1994;54:1587–95. 8137264

[29] Long X Nephew KP. Fulvestrant (ICI 182,780)-dependent interacting proteins mediate immobilization and degradation of estrogen receptor-alpha. J Biol Chem. 2006;281:9607–15. 10.1074/jbc.M510809200 16459337

[30] Spring LM Gupta A Reynolds KL Gadd MA Ellisen LW Isakoff SJ Neoadjuvant endocrine therapy for estrogen receptor-positive breast cancer: a systematic review and meta-analysis. JAMA Oncol. 2016;2:1477–86. 10.1001/jamaoncol.2016.1897 27367583 PMC5738656

[31] Mouridsen H Gershanovich M Sun Y Pérez-Carrión R Boni C Monnier A Superior efficacy of letrozole *versus* tamoxifen as first-line therapy for postmenopausal women with advanced breast cancer: results of a phase III study of the International Letrozole Breast Cancer Group. J Clin Oncol. 2001;19:2596–606. 10.1200/JCO.2001.19.10.2596 11352951

[32] Fekete M Wittliff JL Schally AV. Characteristicsanddistributionofreceptorsfor[D-TRP6]-luteinizing hormone-releasing hormone, somatostatin, epidermal growth factor, and sex steroids in 500 biopsy samples of human breast cancer. J Clin Lab Anal. 1989;3:137–47. 10.1002/jcla.1860030302 2569034

[33] Seitz S Buchholz S Schally AV Weber F Klinkhammer-Schalke M Inwald EC Triple negative breast cancers express receptors for LHRH and are potential therapeutic targets for cytotoxic LHRH-analogs, AEZS 108 and AEZS 125. BMC Cancer. 2014;14:847. 10.1186/1471-2407-14-847 25410881 PMC4289186

[34] Huerta-Reyes M Maya-Núñez G Pérez-Solis MA López-Muñoz E Guillén N Olivo-Marin JC Treatment of breast cancer with gonadotropin-releasing hormone analogs. Front Oncol. 2019;9:943. 10.3389/fonc.2019.00943 31632902 PMC6779786

[35] Lambertini M Ceppi M Poggio F Peccatori FA Azim HA Jr Ugolini D Ovarian suppression using luteinizing hormone-releasing hormone agonists during chemotherapy to preserve ovarian function and fertility of breast cancer patients: a meta-analysis of randomized studies. Ann Oncol. 2015;26:2408–19. 10.1093/annonc/mdv374 26347105

[36] Schirrmacher V. From chemotherapy to biological therapy: a review of novel concepts to reduce the side effects of systemic cancer treatment (review). Int J Oncol. 2019;54:407–19. 10.3892/ijo.2018.4661 30570109 PMC6317661

[37] Hargis JB Nakajima ST. Resumption of menses with initiation of letrozole after five years of amenorrhea on tamoxifen: caution needed when using tamoxifen followed by aromatase inhibitors. Cancer Invest. 2006;24:174–7. 10.1080/07357900500524538 16537187

[38] Augereau P Patsouris A Bourbouloux E Gourmelon C Abadie Lacourtoisie S Berton Rigaud D Hormonoresistance in advanced breast cancer: a new revolution in endocrine therapy. Ther Adv Med Oncol. 2017;9:335–46. 10.1177/1758834017693195 28529550 PMC5424863

[39] Jeselsohn R Buchwalter G De Angelis C Brown M Schiff R. *ESR1* mutations—a mechanism for acquired endocrine resistance in breast cancer. Nat Rev Clin Oncol. 2015;12:573–83. 10.1038/nrclinonc.2015.117 26122181 PMC4911210

[40] Gutierrez MC Detre S Johnston S Mohsin SK Shou J Allred DC Molecular changes in tamoxifen-resistant breast cancer: relationship between estrogen receptor, HER-2, and p38 mitogen-activated protein kinase. J Clin Oncol. 2005;23:2469–76. 10.1200/JCO.2005.01.172 15753463

[41] Yang X Ferguson AT Nass SJ Phillips DL Butash KA Wang SM Transcriptional activation of estrogen receptor alpha in human breast cancer cells by histone deacetylase inhibition. Cancer Res. 2000;60:6890–4. 11156387

[42] Ottaviano YL Issa JP Parl FF Smith HS Baylin SB Davidson NE. Methylation of the estrogen receptor gene CpG island marks loss of estrogen receptor expression in human breast cancer cells. Cancer Res. 1994;54:2552–5. 8168078

[43] Toy W Weir H Razavi P Lawson M Goeppert AU Mazzola AM Activating *ESR1* mutations differentially affect the efficacy of ER antagonists. Cancer Discov. 2017;7:277–87. 10.1158/2159-8290.CD-15-1523 27986707 PMC5340622

[44] Toy W Shen Y Won H Green B Sakr RA Will M *ESR1* ligand-binding domain mutations in hormone-resistant breast cancer. Nat Genet. 2013;45:1439–45. 10.1038/ng.2822 24185512 PMC3903423

[45] Reinert T Saad ED Barrios CH Bines J. Clinical implications of ESR1 mutations in hormone receptor-positive advanced breast cancer. Front Oncol. 2017;7:26. 10.3389/fonc.2017.00026 28361033 PMC5350138

[46] Jeselsohn R De Angelis C Brown M Schiff R. The evolving role of the estrogen receptor mutations in endocrine therapy-resistant breast cancer. Curr Oncol Rep. 2017;19:35. 10.1007/s11912-017-0591-8 28374222

[47] Thomas C Gustafsson JÅ. Estrogen receptor mutations and functional consequences for breast cancer. Trends Endocrinol Metab. 2015;26:467–76. 10.1016/j.tem.2015.06.007 26183887

[48] Alayev A Salamon RS Berger SM Schwartz NS Cuesta R Snyder RB mTORC1 directly phosphorylates and activates ERα upon estrogen stimulation. Oncogene. 2016;35:3535–43. 10.1038/onc.2015.414 26522726 PMC4853282

[49] Katzenellenbogen JA Mayne CG Katzenellenbogen BS Greene GL Chandarlapaty S. Structural underpinnings of oestrogen receptor mutations in endocrine therapy resistance. Nat Rev Cancer. 2018;18:377–88. Erratum in: Nat Rev Cancer. 2018;18:662. 10.1038/s41568-018-0053-0 29662238 PMC6252060

[50] Arnesen S Blanchard Z Williams MM Berrett KC Li Z Oesterreich S Estrogen receptor alpha mutations in breast cancer cells cause gene expression changes through constant activity and secondary effects. Cancer Res. 2021;81:539–51. 10.1158/0008-5472.CAN-20-1171 33184109 PMC7854489

[51] Jeselsohn R Bergholz JS Pun M Cornwell M Liu W Nardone A Allele-specific chromatin recruitment and therapeutic vulnerabilities of ESR1 activating mutations. Cancer Cell. 2018;33:173–86.e5. 10.1016/j.ccell.2018.01.004 29438694 PMC5813700

[52] Hartmaier RJ Trabucco SE Priedigkeit N Chung JH Parachoniak CA Vanden Borre P Recurrent hyperactive *ESR1* fusion proteins in endocrine therapy-resistant breast cancer. Ann Oncol. 2018;29:872–80. 10.1093/annonc/mdy025 29360925 PMC5913625

[53] Li L Lin L Veeraraghavan J Hu Y Wang X Lee S Therapeutic role of recurrent *ESR1*-CCDC170 gene fusions in breast cancer endocrine resistance. Breast Cancer Res. 2020;22:84. 10.1186/s13058-020-01325-3 32771039 PMC7414578

[54] Jeong JH Yun JW Kim HY Heo CY Lee S. Elucidation of novel therapeutic targets for breast cancer with *ESR1-CCDC170* fusion. J Clin Med. 2021;10:582. 10.3390/jcm10040582 33557149 PMC7913953

[55] Fanning SW Greene GL. Next-generation ERα inhibitors for endocrine-resistant ER^+^ breast cancer. Endocrinology. 2019;160:759–69. 10.1210/en.2018-01095 30753408

[56] Bardia A Kaklamani V Wilks S Weise A Richards D Harb W Phase I study of elacestrant (RAD1901), a novel selective estrogen receptor degrader, in ER-positive, HER2-negative advanced breast cancer. J Clin Oncol. 2021;39:1360–70. 10.1200/JCO.20.02272 33513026 PMC8078341

[57] Jhaveri K Juric D Yap YS Cresta S Layman RM Duhoux FP A phase I study of LSZ102, an oral selective estrogen receptor degrader, with or without ribociclib or alpelisib, in patients with estrogen receptor-positive breast cancer. Clin Cancer Res. 2021;27:5760–70. 10.1158/1078-0432.CCR-21-1095 34433648 PMC9401512

[58] Savas P Salgado R Denkert C Sotiriou C Darcy PK Smyth MJ Clinical relevance of host immunity in breast cancer: from TILs to the clinic. Nat Rev Clin Oncol. 2016;13:228–41. 10.1038/nrclinonc.2015.215 26667975

[59] Yates LR Knappskog S Wedge D Farmery JHR Gonzalez S Martincorena I Genomic evolution of breast cancer metastasis and relapse. Cancer Cell. 2017;32:169–84.e7. 10.1016/j.ccell.2017.07.005 28810143 PMC5559645

[60] Lee AV Cui X Oesterreich S. Cross-talk among estrogen receptor, epidermal growth factor, and insulin-like growth factor signaling in breast cancer. Clin Cancer Res. 2001;7:4429s–35s; discussion 4411s–2s. 11916236

[61] Font de Mora J Brown M. AIB1 is a conduit for kinase-mediated growth factor signaling to the estrogen receptor. Mol Cell Biol. 2000;20:5041–7. 10.1128/MCB.20.14.5041-5047.2000 10866661 PMC85954

[62] Campbell RA Bhat-Nakshatri P Patel NM Constantinidou D Ali S Nakshatri H. Phosphatidylinositol 3-kinase/AKT-mediated activation of estrogen receptor alpha: a new model for anti-estrogen resistance. J Biol Chem. 2001;276:9817–24. 10.1074/jbc.M010840200 11139588

[63] Arpino G Wiechmann L Osborne CK Schiff R. Crosstalk between the estrogen receptor and the HER tyrosine kinase receptor family: molecular mechanism and clinical implications for endocrine therapy resistance. Endocr Rev. 2008;29:217–33. 10.1210/er.2006-0045 18216219 PMC2528847

[64] Lupien M Meyer CA Bailey ST Eeckhoute J Cook J Westerling T Growth factor stimulation induces a distinct ERα cistrome underlying breast cancer endocrine resistance. Genes Dev. 2010;24:2219–27. 10.1101/gad.1944810 20889718 PMC2947773

[65] Mao P Cohen O Kowalski KJ Kusiel JG Buendia-Buendia JE Cuoco MS Acquired FGFR and FGF alterations confer resistance to estrogen receptor (ER) targeted therapy in ER^+^ metastatic breast cancer. Clin Cancer Res. 2020;26:5974–89. 10.1158/1078-0432.CCR-19-3958 32723837

[66] Tomlinson DC Knowles MA Speirs V. Mechanisms of FGFR3 actions in endocrine resistant breast cancer. Int J Cancer. 2012;130:2857–66. 10.1002/ijc.26304 21792889

[67] Autier P Boniol M La Vecchia C Vatten L Gavin A Héry C Disparities in breast cancer mortality trends between 30 European countries: retrospective trend analysis of WHO mortality database. BMJ. 2010;341:c3620. Erratum in: BMJ. 2010;341:c4480. 10.1136/bmj.c3620 20702548 PMC2920378

[68] Bosch A Li Z Bergamaschi A Ellis H Toska E Prat A PI3K inhibition results in enhanced estrogen receptor function and dependence in hormone receptor-positive breast cancer. Sci Transl Med. 2015;7:283ra51. Erratum in: Sci Transl Med. 2018;10:eaav7516. 10.1126/scitranslmed.aaa4442 25877889 PMC4433148

[69] Ribas R Pancholi S Guest SK Marangoni E Gao Q Thuleau A AKT antagonist AZD5363 influences estrogen receptor function in endocrine-resistant breast cancer and synergizes with fulvestrant (ICI182780) *in vivo*. Mol Cancer Ther. 2015;14:2035–48. 10.1158/1535-7163.MCT-15-0143 26116361

[70] Croessmann S Formisano L Kinch LN Gonzalez-Ericsson PI Sudhan DR Nagy RJ Combined blockade of activating *ERBB2* mutations and ER results in synthetic lethality of ER+/HER2 mutant breast cancer. Clin Cancer Res. 2019;25:277–89. 10.1158/1078-0432.CCR-18-1544 30314968 PMC6320312

[71] Smyth LM Piha-Paul SA Won HH Schram AM Saura C Loi S Efficacy and determinants of response to HER kinase inhibition in *HER2*-mutant metastatic breast cancer. Cancer Discov. 2020;10:198–213. 10.1158/2159-8290.CD-19-0966 31806627 PMC7007377

[72] Robinson DR Wu YM Lin SF. The protein tyrosine kinase family of the human genome. Oncogene. 2000;19:5548–57. 10.1038/sj.onc.1203957 11114734

[73] Templeton AJ Diez-Gonzalez L Ace O Vera-Badillo F Seruga B Jordán J Prognostic relevance of receptor tyrosine kinase expression in breast cancer: a meta-analysis. Cancer Treat Rev. 2014;40:1048–55. 10.1016/j.ctrv.2014.08.003 25217796

[74] Tomiguchi M Yamamoto Y Yamamoto-Ibusuki M Goto-Yamaguchi L Fujiki Y Fujiwara S Fibroblast growth factor receptor-1 protein expression is associated with prognosis in estrogen receptor-positive/human epidermal growth factor receptor-2-negative primary breast cancer. Cancer Sci. 2016;107:491–8. 10.1111/cas.12897 26801869 PMC4832856

[75] Ocaña A Amir E Seruga B Martin M Pandiella A. The evolving landscape of protein kinases in breast cancer: clinical implications. Cancer Treat Rev. 2013;39:68–76. 10.1016/j.ctrv.2012.05.004 22703833

[76] Lemmon MA Schlessinger J. Cell signaling by receptor tyrosine kinases. Cell. 2010;141:1117–34. 10.1016/j.cell.2010.06.011 20602996 PMC2914105

[77] Linggi B Carpenter G. ErbB receptors: new insights on mechanisms and biology. Trends Cell Biol. 2006;16:649–56. 10.1016/j.tcb.2006.10.008 17085050

[78] Braicu C Buse M Busuioc C Drula R Gulei D Raduly L A comprehensive review on MAPK: a promising therapeutic target in cancer. Cancers (Basel). 2019;11:1618. 10.3390/cancers11101618 31652660 PMC6827047

[79] Manning BD Cantley LC. AKT/PKB signaling: navigating downstream. Cell. 2007;129:1261–74. 10.1016/j.cell.2007.06.009 17604717 PMC2756685

[80] Yang J Nie J Ma X Wei Y Peng Y Wei X. Targeting PI3K in cancer: mechanisms and advances in clinical trials. Mol Cancer. 2019;18:26. 10.1186/s12943-019-0954-x 30782187 PMC6379961

[81] Ghayad SE Vendrell JA Ben Larbi S Dumontet C Bieche I Cohen PA. Endocrine resistance associated with activated ErbB system in breast cancer cells is reversed by inhibiting MAPK or PI3K/Akt signaling pathways. Int J Cancer. 2010;126:545–62. 10.1002/ijc.24750 19609946

[82] Kim EK Choi EJ. Pathological roles of MAPK signaling pathways in human diseases. Biochim Biophys Acta. 2010;1802:396–405. 10.1016/j.bbadis.2009.12.009 20079433

[83] Zheng Y Sowers JY Houston KD. IGFBP-1 expression promotes tamoxifen resistance in breast cancer cells via Erk pathway activation. Front Endocrinol (Lausanne). 2020;11:233. 10.3389/fendo.2020.00233 32435229 PMC7218143

[84] Chen D Washbrook E Sarwar N Bates GJ Pace PE Thirunuvakkarasu V Phosphorylation of human estrogen receptor alpha at serine 118 by two distinct signal transduction pathways revealed by phosphorylation-specific antisera. Oncogene. 2002;21:4921–31. 10.1038/sj.onc.1205420 12118371

[85] Bayliss J Hilger A Vishnu P Diehl K El-Ashry D. Reversal of the estrogen receptor negative phenotype in breast cancer and restoration of antiestrogen response. Clin Cancer Res. 2007;13:7029–36. 10.1158/1078-0432.CCR-07-0587 18056179

[86] Zhang XT Kang LG Ding L Vranic S Gatalica Z Wang ZY. A positive feedback loop of ER-α36/EGFR promotes malignant growth of ER-negative breast cancer cells. Oncogene. 2011;30:770–80. 10.1038/onc.2010.458 20935677 PMC3020987

[87] Lappano R De Marco P De Francesco EM Chimento A Pezzi V Maggiolini M. Cross-talk between GPER and growth factor signaling. J Steroid Biochem Mol Biol. 2013;137:50–6. 10.1016/j.jsbmb.2013.03.005 23542661

[88] Filardo EJ Thomas P. Minireview: G protein-coupled estrogen receptor-1, GPER-1: its mechanism of action and role in female reproductive cancer, renal and vascular physiology. Endocrinology. 2012;153:2953–62. 10.1210/en.2012-1061 22495674 PMC3380306

[89] Luo J Liu D. Does GPER really function as a G protein-coupled estrogen receptor *in vivo*? Front Endocrinol (Lausanne). 2020;11:148. 10.3389/fendo.2020.00148 32296387 PMC7137379

[90] Revankar CM Cimino DF Sklar LA Arterburn JB Prossnitz ER. A transmembrane intracellular estrogen receptor mediates rapid cell signaling. Science. 2005;307:1625–30. 10.1126/science.1106943 15705806

[91] Filardo EJ Quinn JA Bland KI Frackelton AR Jr. Estrogen-induced activation of Erk-1 and Erk-2 requires the G protein-coupled receptor homolog, GPR30, and occurs via trans-activation of the epidermal growth factor receptor through release of HB-EGF. Mol Endocrinol. 2000;14:1649–60. 10.1210/mend.14.10.0532 11043579

[92] Mo Z Liu M Yang F Luo H Li Z Tu G GPR30 as an initiator of tamoxifen resistance in hormone-dependent breast cancer. Breast Cancer Res. 2013;15:R114. 10.1186/bcr3581 24289103 PMC3978564

[93] Jhanwar-Uniyal M Wainwright JV Mohan AL Tobias ME Murali R Gandhi CD Diverse signaling mechanisms of mTOR complexes: mTORC1 and mTORC2 in forming a formidable relationship. Adv Biol Regul. 2019;72:51–62. 10.1016/j.jbior.2019.03.003 31010692

[94] Alayev A Holz MK. mTOR signaling for biological control and cancer. J Cell Physiol. 2013;228:1658–64. 10.1002/jcp.24351 23460185 PMC4491917

[95] Loewith R Jacinto E Wullschleger S Lorberg A Crespo JL Bonenfant D Two TOR complexes, only one of which is rapamycin sensitive, have distinct roles in cell growth control. Mol Cell. 2002;10:457–68. 10.1016/S1097-2765(02)00636-6 12408816

[96] Porta C Paglino C Mosca A. Targeting PI3K/Akt/mTOR signaling in cancer. Front Oncol. 2014;4:64. 10.3389/fonc.2014.00064 24782981 PMC3995050

[97] Hare SH Harvey AJ. mTOR function and therapeutic targeting in breast cancer. Am J Cancer Res. 2017;7:383–404. 28400999 PMC5385631

[98] Jordan NJ Dutkowski CM Barrow D Mottram HJ Hutcheson IR Nicholson RI Impact of dual mTORC1/2 mTOR kinase inhibitor AZD8055 on acquired endocrine resistance in breast cancer *in vitro*. Breast Cancer Res. 2014;16:R12. 10.1186/bcr3604 24457069 PMC3978713

[99] Alayev A Berger SM Kramer MY Schwartz NS Holz MK. The combination of rapamycin and resveratrol blocks autophagy and induces apoptosis in breast cancer cells. J Cell Biochem. 2015;116:450–7. 10.1002/jcb.24997 25336146 PMC4491987

[100] Pérez-Tenorio G Karlsson E Waltersson MA Olsson B Holmlund B Nordenskjöld B Clinical potential of the mTOR targets S6K1 and S6K2 in breast cancer. Breast Cancer Res Treat. 2011;128:713–23. 10.1007/s10549-010-1058-x 20953835

[101] Bostner J Alayev A Berman AY Fornander T Nordenskjöld B Holz MK Raptor localization predicts prognosis and tamoxifen response in estrogen receptor-positive breast cancer. Breast Cancer Res Treat. 2018;168:17–27. 10.1007/s10549-017-4508-x 29128895 PMC5847064

[102] Mazumder A Shiao S Haricharan S. HER2 activation and endocrine treatment resistance in HER2-negative breast cancer. Endocrinology. 2021;162:bqab153. 10.1210/endocr/bqab153 34320193 PMC8379900

[103] Houssami N Macaskill P Balleine RL Bilous M Pegram MD. HER2 discordance between primary breast cancer and its paired metastasis: tumor biology or test artefact? Insights through meta-analysis. Breast Cancer Res Treat. 2011;129:659–74. 10.1007/s10549-011-1632-x 21698410

[104] Nair BC Vadlamudi RK. Regulation of hormonal therapy resistance by cell cycle machinery. Gene Ther Mol Biol. 2008;12:395. 20148177 PMC2817953

[105] Sherr CJ Beach D Shapiro GI. Targeting CDK4 and CDK6: from discovery to therapy. Cancer Discov. 2016;6:353–67. 10.1158/2159-8290.CD-15-0894 26658964 PMC4821753

[106] Butt AJ McNeil CM Musgrove EA Sutherland RL. Downstream targets of growth factor and oestrogen signalling and endocrine resistance: the potential roles of c-Myc, cyclin D1 and cyclin E. Endocr Relat Cancer. 2005;12:S47–59. 10.1677/erc.1.00993 16113099

[107] Finn RS Martin M Rugo HS Jones S Im SA Gelmon K Palbociclib and letrozole in advanced breast cancer. N Engl J Med. 2016;375:1925–36. 10.1056/NEJMoa1607303 27959613

[108] Turner NC Slamon DJ Ro J Bondarenko I Im SA Masuda N Overall survival with palbociclib and fulvestrant in advanced breast cancer. N Engl J Med. 2018;379:1926–36. 10.1056/NEJMoa1810527 30345905

[109] O’Brien NA McDermott MSJ Conklin D Luo T Ayala R Salgar S Targeting activated PI3K/mTOR signaling overcomes acquired resistance to CDK4/6-based therapies in preclinical models of hormone receptor-positive breast cancer. Breast Cancer Res. 2020;22:89. 10.1186/s13058-020-01320-8 32795346 PMC7427086

[110] Giltnane JM Hutchinson KE Stricker TP Formisano L Young CD Estrada MV Genomic profiling of ER^+^ breast cancers after short-term estrogen suppression reveals alterations associated with endocrine resistance. Sci Transl Med. 2017;9:eaai7993. Erratum in: Sci Transl Med. 2019;11:eaaw7620. 10.1126/scitranslmed.aai7993 28794284 PMC5723145

[111] Bardia A Hurvitz SA DeMichele A Clark AS Zelnak A Yardley DA Phase I/II trial of exemestane, ribociclib, and everolimus in women with HR^+^/HER2^–^ advanced breast cancer after progression on CDK4/6 inhibitors (TRINITI-1). Clin Cancer Res. 2021;27:4177–85. 10.1158/1078-0432.CCR-20-2114 33722897 PMC8487593

[112] Spring LM Wander SA Andre F Moy B Turner NC Bardia A. Cyclin-dependent kinase 4 and 6 inhibitors for hormone receptor-positive breast cancer: past, present, and future. Lancet. 2020;395:817–27. 10.1016/S0140-6736(20)30165-3 32145796

[113] Sledge GW Jr Toi M Neven P Sohn J Inoue K Pivot X The effect of abemaciclib plus fulvestrant on overall survival in hormone receptor-positive, ERBB2-negative breast cancer that progressed on endocrine therapy-MONARCH 2: a randomized clinical trial. JAMA Oncol. 2020;6:116–24. 10.1001/jamaoncol.2019.4782 31563959 PMC6777264

[114] Cottu P D'Hondt V Dureau S Lerebours F Desmoulins I Heudel PE Letrozole and palbociclib *versus* chemotherapy as neoadjuvant therapy of high-risk luminal breast cancer. Ann Oncol. 2018;29:2334–40. 10.1093/annonc/mdy448 30307466

[115] Wander SA Juric D Supko JG Micalizzi DS Spring L Vidula N Phase Ib trial to evaluate safety and anti-tumor activity of the AKT inhibitor, ipatasertib, in combination with endocrine therapy and a CDK4/6 inhibitor for patients with hormone receptor positive (HR^+^)/HER2 negative metastatic breast cancer (MBC) (TAKTIC). J Clin Oncol. 2020;38:1066. 10.1200/JCO.2020.38.15_suppl.1066

[116] Cancer Genome Atlas Network. Comprehensive molecular portraits of human breast tumours. Nature. 2012;490:61–70. 10.1038/nature11412 23000897 PMC3465532

[117] Goncalves MD Hopkins BD Cantley LC. Phosphatidylinositol 3-kinase, growth disorders, and cancer. N Engl J Med. 2018;379:2052–62. 10.1056/NEJMra1704560 30462943

[118] André F Ciruelos EM Juric D Loibl S Campone M Mayer IA Alpelisib plus fulvestrant for *PIK3CA*-mutated, hormone receptor-positive, human epidermal growth factor receptor-2-negative advanced breast cancer: final overall survival results from SOLAR-1. Ann Oncol. 2021;32:208–17. 10.1016/j.annonc.2020.11.011 33246021

[119] Rinnerthaler G Gampenrieder SP Greil R. HER2 directed antibody-drug-conjugates beyond T-DM1 in breast cancer. Int J Mol Sci. 2019;20:1115. 10.3390/ijms20051115 30841523 PMC6429068

[120] Werner S Heidrich I Pantel K. Clinical management and biology of tumor dormancy in breast cancer. Semin Cancer Biol. 2022;78:49–62. 10.1016/j.semcancer.2021.02.001 33582172

